# Environmental Influences on the Abundance and Sexual Composition of White Sharks *Carcharodon carcharias* in Gansbaai, South Africa

**DOI:** 10.1371/journal.pone.0071197

**Published:** 2013-08-12

**Authors:** Alison V. Towner, Les G. Underhill, Oliver J. D. Jewell, Malcolm J. Smale

**Affiliations:** 1 Dyer Island Conservation Trust, Kleinbaai, South Africa; 2 Animal Demography Unit, Department of Biological Sciences, University of Cape Town, Rondebosch, South Africa; 3 Marine Research Institute, University of Cape Town, Rondebosch, South Africa; 4 Mammal Research Institute, University of Pretoria, Hatfield, Pretoria, South Africa; 5 Port Elizabeth Museum at Bayworld, Humewood, South Africa; 6 Department of Zoology, Nelson Mandela Metropolitan University, Port Elizabeth, South Africa; Aristotle University of Thessaloniki, Greece

## Abstract

The seasonal occurrence of white sharks visiting Gansbaai, South Africa was investigated from 2007 to 2011 using sightings from white shark cage diving boats. Generalized linear models were used to investigate the number of great white sharks sighted per trip in relation to sex, month, sea surface temperature and Multivariate El Niño/Southern Oscillation (ENSO) Indices (MEI). Water conditions are more variable in summer than winter due to wind-driven cold water upwelling and thermocline displacement, culminating in colder water temperatures, and shark sightings of both sexes were higher during the autumn and winter months (March–August). MEI, an index to quantify the strength of Southern Oscillation, differed in its effect on the recorded numbers of male and female white sharks, with highly significant interannual trends. This data suggests that water temperature and climatic phenomena influence the abundance of white sharks at this coastal site. In this study, more females were seen in Gansbaai overall in warmer water/positive MEI years. Conversely, the opposite trend was observed for males. In cool water years (2010 to 2011) sightings of male sharks were significantly higher than in previous years. The influence of environmental factors on the physiology of sharks in terms of their size and sex is discussed. The findings of this study could contribute to bather safety programmes because the incorporation of environmental parameters into predictive models may help identify times and localities of higher risk to bathers and help mitigate human-white shark interactions.

## Introduction

White sharks *Carcharodon carcharias* (Linnaeus 1758) are large apex predators that occur circumglobally in cool temperate marine systems [1]. Until the 1990s they were thought to be a primarily coastal species [2]. Aggregations occur at predictable coastal locations in the USA, Mexico, South Africa, New Zealand and Australia [3]–[7]. The predictability of white sharks at known locations and their apparent site fidelity suggest that they select these locations at specific times of the year [8].

We now know, through advances in tagging technologies, that these predators undertake extensive pelagic migrations, crossing ocean basins and inhabiting tropical waters before returning and exhibiting fidelity to temperate coastal aggregation sites [8]–[16]. While in the tropics and open ocean, they dive to depths exceeding 500 m and as deep as 1,200 m [10], [17]–[21]. Hunting, mating and breeding have been suggested as possible causes of this migratory behaviour [8], [10], [13], [20]–[22]. Furthermore, environmental cues such as changes in water temperature and upwelling influence the time spent in the pelagic and coastal phases of these migrations [8], [11], [14]–[16], [18], [20], [21], [23].

During deep dives in oceanic waters, mature white sharks experience cold and hypoxic waters. Tracking evidence has indicated that they can tolerate lower extremes of 2.5°C and 1.5–2.0 mL/L^–l^ of oxygen [16], [18], [19], [21]. Whilst most fish species return to the surface soon after undertaking dives below the thermocline [24], satellite tagging studies have shown that larger white sharks (>3.5 m TL) are capable of spending longer time periods than most other fish species in these environments, sometimes more than 12 hours, which is possible because of their physiological adaptations and thermoregulation [18], [20], [21], [24]–[29]. White sharks have been tracked into water temperatures of 6.8°C in Australia [15] and into waters of 2.5°C in New Zealand [21] however dives were limited to 10–15 min./dive [16].

Animals make movement decisions to acquire food and mates, evade predators and select appropriate environmental conditions [30]. On returning to temperate coastal areas, white sharks generally dive less frequently and to shallower depths, as they are limited to continental shelf waters [21]. White sharks on the coastal shelf in Australia, California and New Zealand spend most of their time between the surface and 50 m where water temperatures remain more stable [21]. Furthermore, different size classes of white sharks seek out different sites when visiting coastal regions [31]–[33]. These are probably determined by prey availability, suitable environmental conditions, or both. Throughout their range [16], [34], [35] adult and juvenile female white sharks [16] are more frequently documented inshore in summer months than male sharks [31]–[33], [36].

El Niño/Southern Oscillation (ENSO) is the most important coupled ocean-atmosphere phenomenon to cause global climate variability on seasonal to interannual time scales [37]. During warm events, atmospheric pressure rises in the western Pacific Ocean and falls in the eastern Pacific Ocean, weakening or even reversing the direction of south east trade winds [38]. This causes suppression of the thermocline (pushing it deeper), with a pool of warm water surging eastwards along the equator, towards South America and a reduction of the sea level in the western Pacific Ocean [39]–[41]. It is an important component of climate variability along the South African south coast [42], [43]. The periodicity of ENSO events varies between two and ten years [41], [44] with an average return period of three years [45]. Multivariate El Niño/Southern Oscillation (ENSO) Index (MEI) is a multivariate measure of the ENSO using six of the main observed variables over the tropical Pacific Ocean [37]. MEI integrates more information than other indices, such as the SOI (Southern Oscillation Index), which is based on Tahiti-Darwin pressure difference alone. MEI is thought to better reflect the nature of the coupled ocean-atmosphere system compared to other indices [38]. The highest values of MEI represent the warm ENSO phase (El Niño) while the lowest values of MEI represent the cold ENSO phase (La Niña). Furthermore, the minima and maxima of MEI follow a 60 month cycle [46]. In South Africa, ENSO has been studied in detail particularly in relation to commercially important fishery species such as anchovy, sardine and squid [47], [48]. Environmental factors have been suggested to explain the higher capture rate of juvenile male white sharks in the anti-shark nets off the KwaZulu-Natal coast during a positive SOI or La Niña cool season [49].

ENSO events occur frequently each decade causing short to medium-term fluctuations in the climate [50]. During these periods, anomalous physical conditions impact widely upon marine biological systems; both positive and negative anomalies affect the ecosystem [43], [51]. While changes in top predator population dynamics have been linked to large scale oceanographic processes in most major ocean basins, the effects are particularly evident where predator species are not able to respond by switching to other prey species [51]. The relationship between these climatic processes and trophic responses is often complex and may be delayed in response to the primary climate signal change. These effects may manifest in predator population distributions, movements, densities, phenology, behaviour and community interactions [51].

Gansbaai, in the Western Cape Province of South Africa, is visited seasonally by various size classes and both sexes of white sharks [33], [52]. In summer, intensified south easterly trade winds result in upwelling [53] causing cold water of Benguela origin to enter the bay. In winter, the westerly wind belt moves northwards and becomes the dominant prevailing wind, reducing upwelling and increasing the leakage of warmer water of Agulhas Bank origin into the bay [54]. The variability of water temperature with season, (upwelling i.e. colder and more variable water temperatures in summer) along with changing environmental parameters such as swell height, wind speed and turbidity between seasons, makes Gansbaai an ideal study site to monitor the influence of environmental parameters on white shark numbers.

This study investigates the seasonal occurrence of white sharks visiting Gansbaai over a five-year period using sighting data recorded from shark cage diving boats. Environmental parameters that may influence the number of white sharks and the sexual composition of the population of animals in the bay are investigated. The relationship of interannual variability in sightings to ENSO events that occurred during the study was also investigated.

## Methods

**Figure 1:**
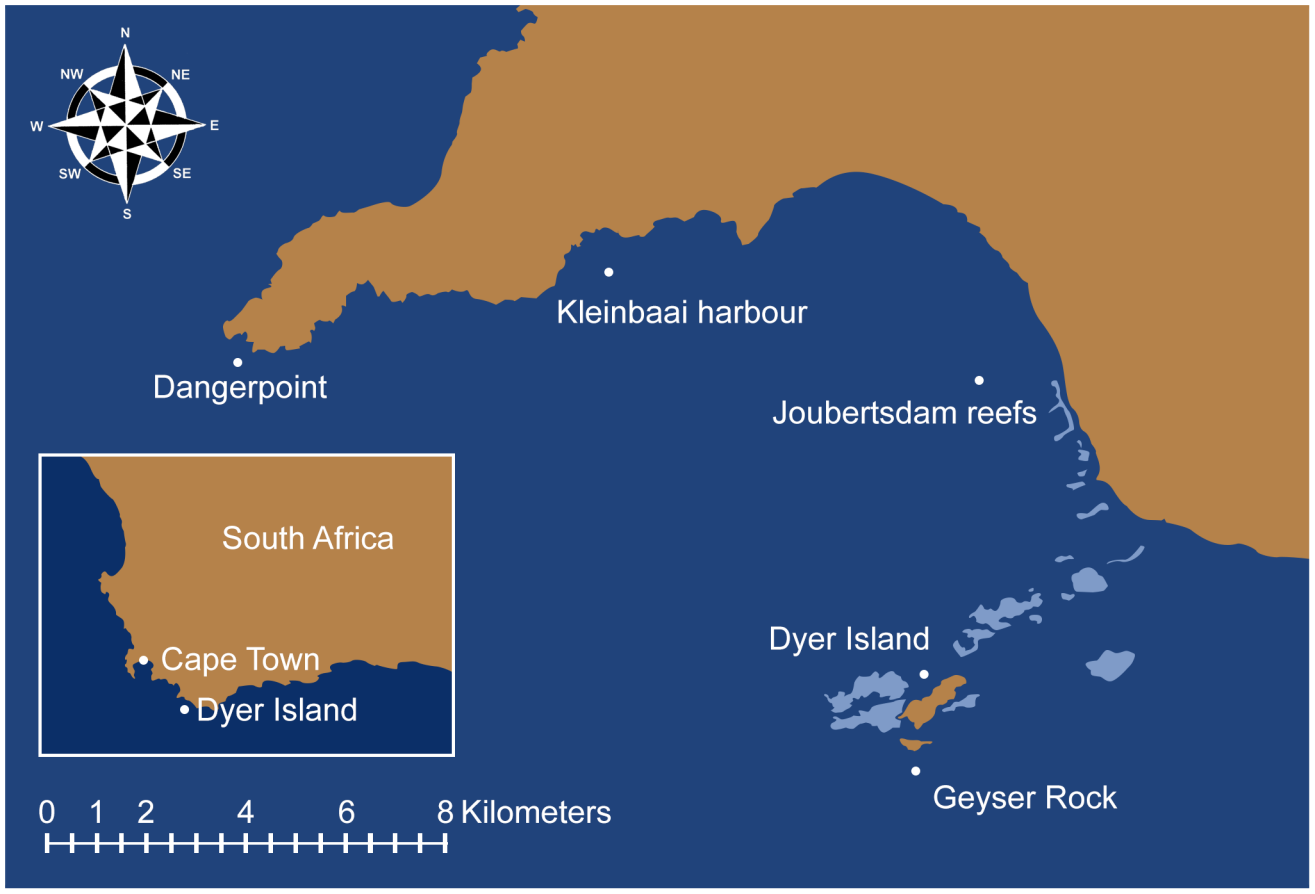
Site Map showing the inshore and island region of Gansbaai. This image was created by EDNA Interactive Ltd trading as EDNA: www.edna.uk.net.

Gansbaai is a semi-enclosed embayment situated on the south coast of the Western Cape, South Africa. It is relatively exposed with the western and eastern boundaries being Danger Point (34°37.50′S; 19°17.30′E) and Quoin Point (34°47.28′S; 19°39.15′E), respectively. White sharks were observed in this study area at two distinct localities: around the periphery of Dyer Island (34°40.669′S, 19°23.863′E) which is located 8 km offshore of the nearest harbour town (Kleinbaai); and at Joubertsdam (34°38.366′S; 19°25.158′E), an inshore reef system running parallel to a sandy beach where sharks occurred in areas as shallow as 2 m deep, immediately behind the surf zone (Figure 1).

During this study, data were collected onboard purpose-built shark cage diving vessels owned by Marine Dynamics, a commercial shark cage diving operator. Between January 2007 and September 2010 a 10.8 m catamaran was used, and from October 2010 until the end of the study in December 2011 a 12 m catamaran was used. Daily log sheets were used to record data on individual sharks and environmental parameters were recorded on every trip. All observations were made from a platform 1 m above sea level. Chumming was initiated on arrival after anchoring at the site at which sharks were expected to be found. Shark cage diving boats operate in these two distinct areas in the bay (Dyer Island and Joubertsdam) and the use of these areas is seasonal. Chumming was undertaken by pouring a mixture of sardine oils and mashed teleost-based chum into the water to create a scent trail on the surface which would orientate and attract the sharks towards the boat [36].

The anchoring position and water depth were recorded using the boat’s navigational equipment, a Furuno GPS (model GP32). Sea surface temperature (SST, measured to the nearest 0.1°C) was recorded during 2007 to 2009 using a probe located 2 m below sea surface in the tunnel of the hull. A Sea-Bird conductivity temperature depth meter (CTD) and a Yellow Springs Instruments (YSI) probe were used to measure these parameters during 2010 to 2011. These instruments were calibrated with the previously used boat sensors to maintain consistency. Wind and current directions were determined from the direction of the chum line, a clearly visible slick on the surface. Underwater visibility (m) was estimated using marked reference points on a dive cage. Swell height (m), sea condition (classified as swell, wind chop or calm) and percentage cloud cover (nearest 10%) were recorded on anchoring. Wind speed (knots) and direction (16 compass points) were obtained from a local weather buoy located offshore of Franskraal (34°38.329′S; 19°25.440′E).

The sex of each shark was determined, if possible, by surface observations and underwater video records of the pelvic fin area. Males were recorded only if claspers were seen. Females were recorded when their pelvic fin area was filmed and the lack of claspers verified, otherwise sharks were recorded as being of unknown sex. Shark size was estimated as individuals passed the measured dive cage, similar to the methods of Kock et al. [33].

Generalized Linear Models with the Poisson distribution were fitted to counts of great white sharks per trip. The variables described above were used as explanatory variables in these models. In addition, long-term trend was measured in months since the start of data collection in January 2007. Models were selected using the Akaike Information Criterion (AIC). Models were fitted for the total number of sharks per trip (males, females and unknown sex), for the number of sharks classified as male and for the number of sharks classified as female. MEI bi-monthly values were incorporated into the GLM for the time period between January 2007 to December 2011. MEI data are tabulated and accessible to the public for download by Klaus Wolter of the National Organisation of Air and Atmospheric Processes (www.esri.noaa.gov/psd/enso/mei/table.html). Both MEI and SOI were initially tested in our Generalized Linear Model. MEI showed the best fit to the results thus was selected as the preferred data for this analysis.

Marine Dynamics holds a commercial cage diving permit issued by the Department of Environmental Affairs: Oceans and Coasts. This study was non-invasive and complied with all relevant laws in South Africa.

## Results

A total of 1,647 trips were made over the five year study period, 923 to offshore island sites and 724 to inshore sites (Table 1, Figure 1). Trips were made to offshore sites mainly from March to August (spanning austral winter) and offshore trips were made in all months except December (early summer). Inshore trips took place mainly during summer, from October to February; no inshore trips were made in April, May and June (winter). During September, which is a month of seasonal transition, 77 offshore trips and 65 inshore trips took place (Table 1, Figure 1).

**Table 1:** Summary of the total number of shark cage-diving trips per month, inshore and offshore, between January 2007 and December 2011 in Gansbaai, South Africa.

Month	Offshore	Inshore	Total
January	29	104	133
February	42	107	149
March	124	35	159
April	124	0	124
May	95	0	95
June	113	0	113
July	123	28	151
August	116	23	139
September	77	65	142
October	50	119	169
November	30	134	164
December	0	109	109
**Totals**	**923**	**724**	**1647**

The mean number of sharks per trip varied from 4.0 sharks in January (summer) to 8.4 sharks in May (autumn); from April (autumn) to October (spring), the mean number of sharks per trip exceeded 6.0 (Table 2). The mean maxima for males and females were both in May (autumn) and July (winter 2.5 and 3.7 sharks/trip respectively) however, the mean minima for males was in October (spring) and for females in February (summer 1.0 and 1.7 sharks/trip respectively) (Table 2). The overall annual mean sea surface temperature was 14.9°C, and the monthly means did not show any strong annual pattern of seasonality although the larger standard deviation values from November (spring) to February (summer) are a consequence of large temperature fluctuations resulting from upwelling (Table 2).

**Table 2:** Mean numbers of white sharks sighted per month (total, female and male) with mean monthly sea surface temperatures in Gansbaai, 2007 to 2011.

Month	Mean Total No. Sharks	S.D	Mean No. Females	S.D	Mean No. Males	S.D	Mean SST°C	S.D
**January**	3.977	2.17	2.204	1.64	1.293	1.46	14.2	2.06
**February**	4.557	3.2	1.746	1.77	1.339	1.35	15.43	2.68
**March**	5.289	3.48	1.977	1.63	1.915	1.46	13.72	1.86
**April**	6.919	4	2.762	1.64	1.845	1.45	13.45	1.93
**May**	8.411	4.32	3.766	2.5	2.598	1.79	14.94	1.43
**June**	7.69	4.51	3.452	2.5	2.337	1.95	15.16	1.1
**July**	7.172	3.84	3.521	2.76	1.607	1.47	14.52	0.62
**August**	7.094	3.66	3.207	2.51	1.613	1.46	14.65	1.05
**September**	6.486	3.22	2.879	2.43	1.224	1.32	15.3	1.36
**October**	6.041	3.21	2.729	2.05	0.991	1.17	15.67	1.89
**November**	5.902	3.16	3.178	2.41	1.719	2.18	16.19	2.7
**December**	5.495	2.81	2.646	1.62	1.192	0.75	14.64	2.2

The sum of the numbers for males and females for each month does not add to the total number seen because the gender of some sharks could not be determined (see text).

Sharks varied in size from <2.0–4.5 m TL (for all sharks, females and males). The size range included juvenile, sub adult and adult males, but only juvenile and sub adult females because females mature at 4.5–5 m length [1] (Table 3). The mature female sharks only made up some 1% of sightings in Gansbaai during this sampling period [55]. Juvenile white sharks are difficult to sex as the male claspers are small, especially when observed in turbid inshore conditions. For all sharks, during spring and summer months, juveniles and larger sub adults sighted inshore were predominantly females and unknown sex. During autumn (March to May) and winter (June to August) sub adults, juveniles and adults of both sexes were sighted at Dyer Island.

**Table 3:** Overall summary statistics of the total lengths (in meters) of white sharks in Gansbaai, January 2007 to December 2011.

	N	Mean	SD	Min	Lower quartile	Median	Upper quartile	Max
**Males**	1929	2.91	0.5	1.5	2.5	2.8	3.2	4.8
**Females**	3671	2.89	0.56	1.6	2.5	2.8	3.2	4.5
**Unsexed**	4663	2.65	0.52	1.5	2.3	2.6	3	5
**All**	10263	2.78	0.55	1.5	2.5	2.7	3	5

Three generalized linear models were fitted: for the total number of sharks per trip which includes males, females, and unknown sex (Table 4), for the number of sharks classified as male (Table 5), and the number of sharks classified as female (Table 6). Preliminary model-building for the three models demonstrated that four of the explanatory variables were important: seasonality (monthly factors), sea surface temperature, long term trend and Multivariate ENSO Index (MEI). The modelled data set for all sharks (Table 7), including these four explanatory variables explained 23.8% of the deviance. Each of the four explanatory variables played an important role in the model. In a conventional modelling approach, all were formally statistically significant (Table 4). The coefficient of the trend variable was positive, indicating an increasing trend in the total numbers of sharks per trip over the five-year study period. The coefficient of the sea surface temperature variable was positive indicating that, over and above the effects of the monthly seasonality factors and MEI, there was a tendency for the number of sharks per trip to be higher when the sea was warmer. Likewise, the coefficient of the MEI was positive, indicating a positive relationship between shark numbers per trip and MEI (more shark sightings during warmer water years).

**Table 4:** Results of a generalized linear model relating total number of great white sharks observed per trip in Gansbaai to specified explanatory variables.

Parameter	Regression Estimate	Standard Error	t(*)	P-value
**January**	0.7685	0.0963	7.98	<.001
**February**	–0.0468	0.0667	–0.70	0.483
**March**	0.1781	0.0617	2.89	0.004
**April**	0.3986	0.0618	6.45	<.001
**May**	0.5729	0.0605	9.47	<.001
**June**	0.521	0.0587	8.88	<.001
**July**	0.4653	0.0564	8.25	<.001
**August**	0.4612	0.0573	8.06	<.001
**September**	0.3879	0.0584	6.64	<.001
**October**	0.2896	0.0581	4.98	<.001
**November**	0.2641	0.0592	4.46	<.001
**December**	0.1941	0.0647	3	0.003
**SST°C**	0.01886	0.00597	3.16	0.002
**Trend**	0.12568	0.00841	14.94	<.001
**MEI**	0.0977	0.0115	8.53	<.001

Model variables included month (with January as the base month), trend (per month), seasonal sea surface temperature (SST) and multivariate ENSO index (MEI).

**Table 5:** Results of a generalized linear model relating total number of male great white sharks observed per trip in Gansbaai to specified explanatory variables.

Parameter	Regression Estimate	Standard Error	t(*)	P-value
**January**	–1.004	0.233	–4.32	<.001
**February**	–0.073	0.157	–0.46	0.642
**March**	0.476	0.133	3.58	<.001
**April**	0.463	0.144	3.23	0.001
**May**	0.842	0.133	6.33	<.001
**June**	0.708	0.131	5.42	<.001
**July**	0.367	0.13	2.82	0.005
**August**	0.324	0.133	2.44	0.015
**September**	–0.081	0.147	–0.55	0.58
**October**	–0.351	0.152	–2.31	0.021
**November**	0.067	0.169	0.4	0.69
**December**	–0.067	0.238	–0.28	0.777
**SST°C**	0.0653	0.0144	4.52	<.001
**Trend**	0.0452	0.0181	2.49	0.013
**MEI**	–0.1083	0.0297	–3.64	<.001

Model variables included month (with January as the base month), trend (per month), seasonal sea surface temperature (SST) and multivariate ENSO index (MEI).

**Table 6:** Results of a generalized linear model relating total number of female great white sharks observed per trip in Gansbaai to specified explanatory variables.

Parameter	Regression Estimate	Standard Error	t(*)	P-value
**January**	1.257	0.158	7.96	<.001
**February**	–0.2290	0.105	–2.18	0.029
**March**	–0.2179	0.0998	–2.18	0.029
**April**	0.0363	0.0984	0.37	0.712
**May**	0.3589	0.0947	3.79	<.001
**June**	0.3038	0.0896	3.39	<.001
**July**	0.3149	0.0844	3.73	<.001
**August**	0.2593	0.0878	2.95	0.003
**September**	0.2863	0.0897	3.19	0.001
**October**	0.2085	0.0894	2.33	0.02
**November**	0.3684	0.0934	3.95	<.001
**December**	0.068	0.119	0.57	0.568
**SST°C**	–0.0238	0.0102	–2.33	0.02
**Trend**	0.0053	0.0137	0.39	0.699
**MEI**	0.2243	0.0188	11.91	<.001

Model variables included month (with January as the base month), trend (per month), seasonal sea surface temperature (SST) and multivariate ENSO index (MEI).

**Table 7:** The results of the generalized linear models for all sharks, males, and females including deviance, Akaike Information Criterion and significance of the variables.

Model	Explanatory Variables	Deviance (%)	AIC	Significant variables
**All sharks**	season, SST, LTT, MEI	23.8	2,354	LTT**, MEI**
**Male sharks**	season, SST, LTT, MEI	12.1	1,418	LTT*, MEI**, SST**
**Female sharks**	season, SST, LTT, MEI	12.9	1,856	MEI**

The explanatory variables were monthly trend (season), sea surface temperature (SST), long term trend (LTT), Multivariate Enso Index (MEI). Significant effects on shark sightings are displayed as (* = p<0.01) and highly significant (** = p<0.001).

For male sharks (Table 5), the model including these four explanatory variables explained 12.1% of the deviance (Table 7). Each explanatory variable was omitted from the model in turn (Table 8). Although the best model for males in relation to explanatory variables accounted for less of the deviance than for the total number of sharks, each of the four explanatory variables was formally statistically significant (Table 5). The coefficient of the trend variable was positive, indicating an increasing trend in the numbers of male sharks per trip over the five-year study period. The coefficient of the sea surface temperature variable was positive. In contrast, the coefficient of the MEI was negative, indicating a statistically significant negative relationship between male shark numbers per trip and MEI. This apparently contradictory finding is discussed below.

**Table 8:** The results of the generalized linear model when specific terms were omitted, for all sharks, females, and males, with the resulting Akaike Information Criterion values shown.

Explanatory Variable Omitted	Deviance (%)	AIC
**All Sharks**		
Season	15.10%	2,596
SST	23.40%	2,362
LTT	16.30%	2,578
MEI	21.40%	2,424
**Male Sharks**		
Season	0.80%	1,574
SST	10.80%	1,437
LTT	11.70%	1,422
MEI	11.20%	1,430
**Female Sharks**		
Season	7.50%	1,961
SST	12.60%	1,872
LTT	12.90%	1,867
MEI	6.20%	2,007

For female sharks (Table 6), the model including these four explanatory variables explained 12.9% of the deviance (Table 7). In the model for females, long term trend and sea surface temperature were not statistically significant explanatory variables (Table 6). In contrast to the model for males (Table 5), the regression coefficient for MEI for females was positive (Table 6).

## Discussion

**Figure 2:**
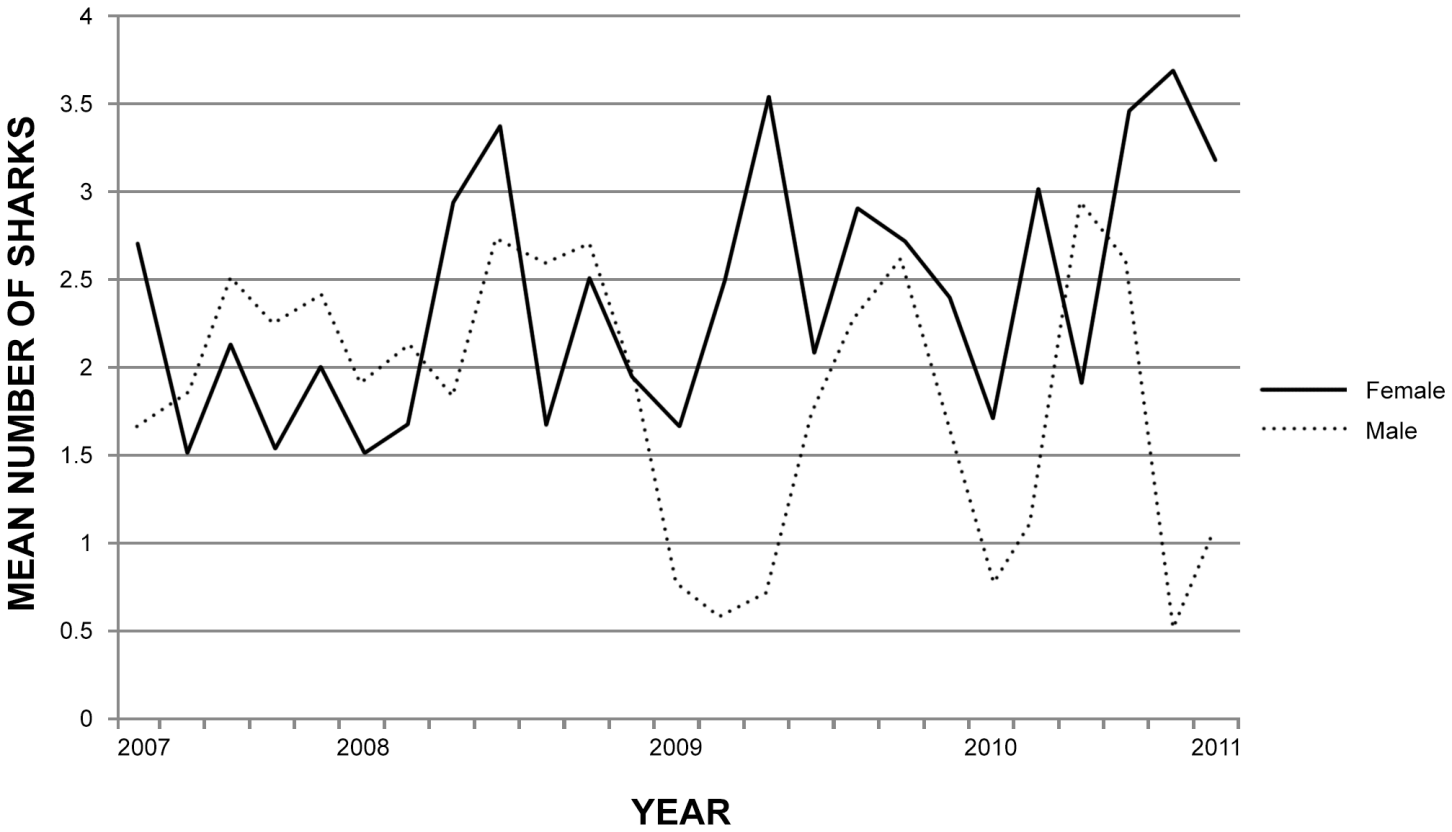
Interannual variability in the mean number of male and female shark sightings per trip at Dyer Island, 2007 to 2011.

**Figure 3:**
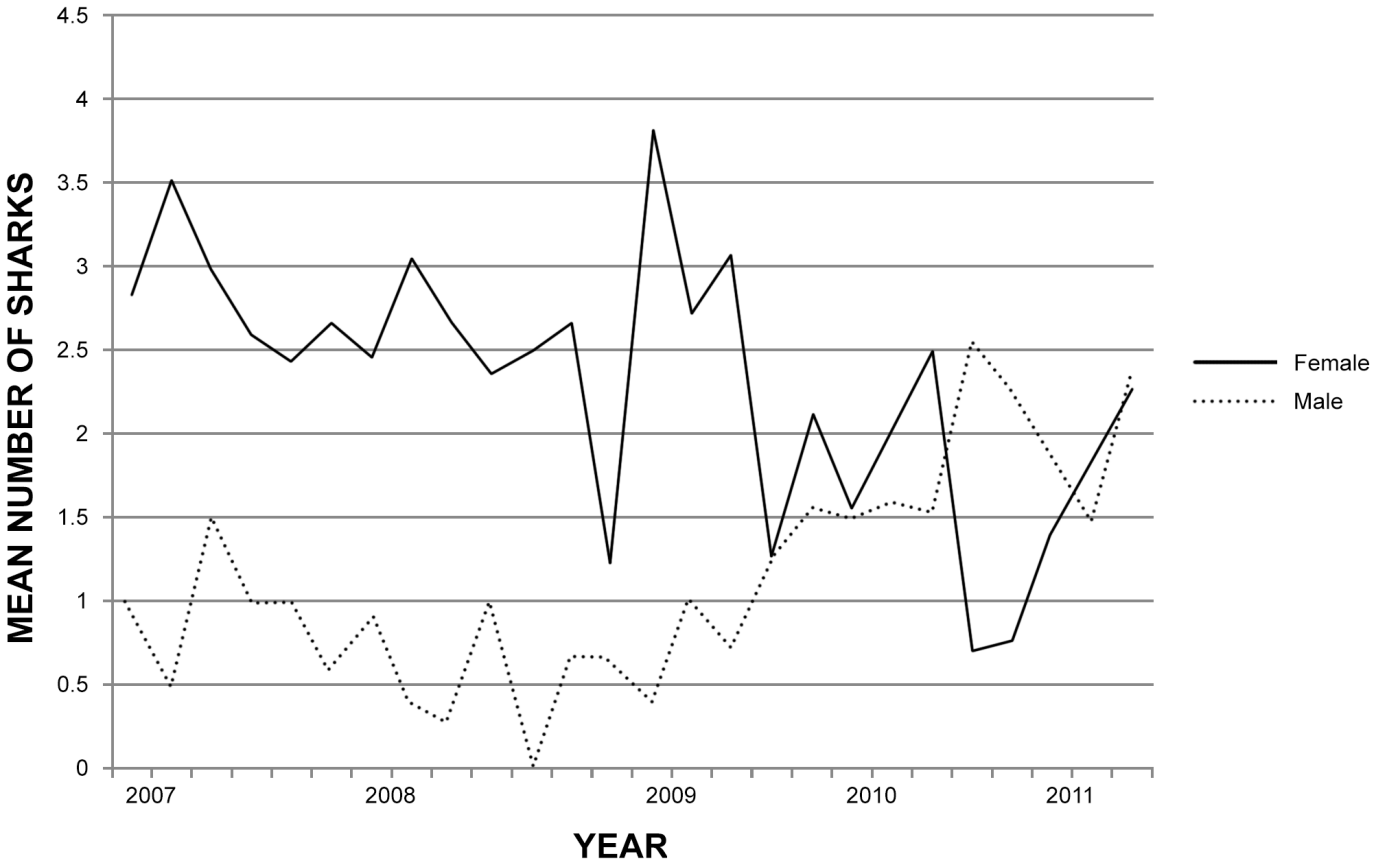
Interannual variability in the mean number of male and female shark sightings per trip at the inshore site in Gansbaai, 2007 to 2011.

**Figure 4:**
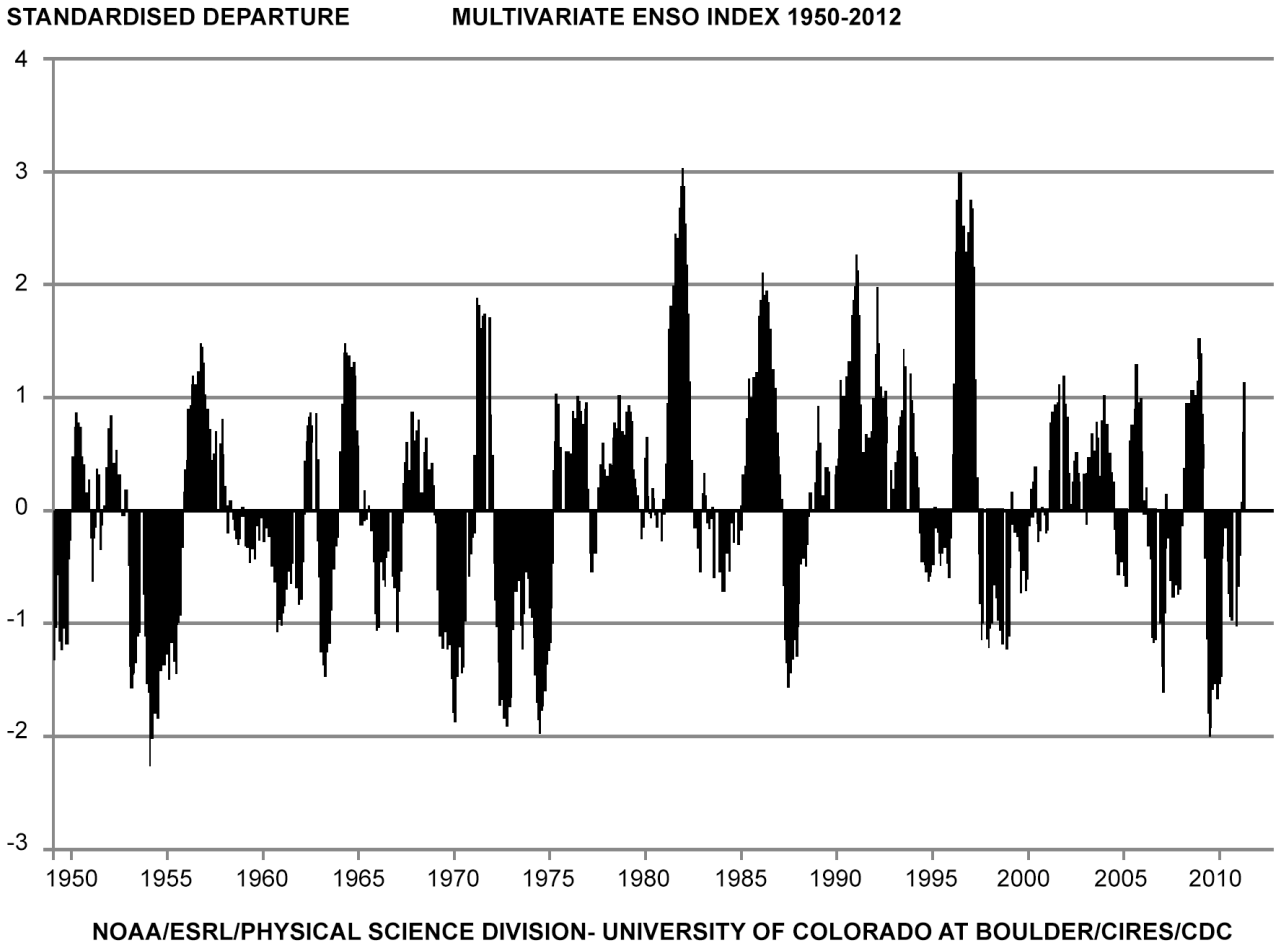
Multivariate ENSO index (MEI) data 1950 to 2010 (Wolter and Timlin 2011 and NOAA). Data sourced from www.noaa.com.

The average number of white shark sightings was higher in autumn and winter months (March to mid-September), at Dyer Island and at Geyser Rock than in summer months (December to February) (Table 1 and 2, Figure 2). During summer months (December to February), sightings were generally female biased when sighting effort shifted to the inshore region of the bay (Tables 1 and 2, Figure 3), with the exception of the summer of 2010 to 2011 when local sea surface temperature was abnormally cold due to increased easterly winds and upwelling resulting from the La Niña event (Figure 4). Co-occurrence of both sexes over several months is also recorded at other white shark aggregation sites at seal colonies located both in coastal sites and offshore islands [11], [19]–[21], [32], [33], [52], [56]. Explanations for the co-occurrence of both sexes at the same location include provisioning on the same prey resources, and in the case of mature animals, possibly for mating purposes [8], [19]. However reproduction is unlikely to be the cause of both sexes co-occurring at Gansbaai, because most female white sharks during this study were too small to be sexually mature (Table 3), [57] and they did not exhibit fresh bites and scarring typical of mating behaviour [58].

Predation by white sharks on Cape fur seals *Arctocephalus pusillus* has been observed at Dyer Island, especially from May to September (autumn and winter) [52], [59]–[62], (AVT unpubl. data). Cape fur seals pup annually and synchronously in November [60]. Yearlings suckle until about five to six months of age, when they start limited foraging in shallow bays in the vicinity of their natal colony [61]. By the age of about seven months (in July to August), the pups start to stay away from the colony for short periods of about three days [63]. The peak in shark sightings for both sexes coincides with a time that may be ideal for hunting inexperienced seals at Dyer Island and the modelled results for all sharks (male, female and unknown sex) in this study indicate that monthly trend was a highly significant explanatory variable. However, when the data was split and modelled for each sex (unknown sex sharks excluded) the monthly trend variable was not significant in some months. This may be a consequence of the smaller samples of the sharks successfully sexed compared to the entire data set including those of unknown sex.

Robbins and Booth [32] suggested that temperature may be an indirect controlling factor on white shark distribution and that white sharks are influenced by one or more other variables that are closely associated with SST. The relationship between female white sharks, SST and monthly trend was not significant when they were modelled separately. The patterns revealed by trends in MEI and SST that appeared to be counterintuitive may be a consequence of a partial mismatch between local conditions at a daily timescale at the chumming site and the regional scale impacts of MEI which occurs over a longer period.

During summer (December to February) the water column is stratified with solar warming of the surface layers. If south-easterly winds blow intensively over a number of days, the warm water is displaced offshore and cold water upwelling occurs. Water temperatures in Gansbaai during austral summer can vary between approximately 9°C and 20°C, with upwelling temperatures decreasing by as much as 10°C within hours or days [64]–[66]. Therefore, water temperature conditions are highly variable during summers. During autumn and winter months (March to August) in South Africa, the westerly wind belt migrates northwards, cyclonic low pressure systems pass south of the African continent and are accompanied by north-westerly winds which push surface waters onshore, and upwelling is minimal [54], [67]. Mixing of the water column occurs, thus, water properties are more stable, particularly in the Agulhas Bank region and in Gansbaai [48], [67]. These more stable winter conditions may be less physiologically stressful to white sharks, possibly making the region more favourable for white sharks over a wide size range, compared to the highly variable thermal conditions experienced in summer. Juvenile and sub adult white sharks are documented more in warmer water temperatures [56] and smaller individuals may be more sensitive to rapid water temperature decreases or to the cooler upwelled water.

A previous study in Australia proposed that pregnant female white sharks may seek out warmer waters to enhance the development of their foetuses [68]. This has been described as the ‘thermal niche hypothesis’. It has also been supported in the north-eastern Pacific Ocean where satellite tagged mature females remained offshore in warmer water temperatures for prolonged time periods, whereas males migrated back to cooler coastal regions in California annually [8], [19]. One 4.8 m female shark was observed to move inshore from an offshore region but made a rapid return offshore before reaching the continental shelf at a time that the tag reported water temperatures at a minimum of 13.6°C [19]. The author suggested that the shark may have been pregnant (from evidence of mating bites when tagged and the large size of the female) and that the cold water may not have been favourable for parturition. To date, the only documented pregnant female white sharks have been recorded during warm water events, positive MEI/El Niño cycles, particularly in the 1990s [69]–[76]. Studies on the reproductive biology of female white sharks in South Africa have been limited by the lack of mature females [57]. The majority of female white sharks encountered in Gansbaai are believed to be sexually immature [57], as supported by information collected in this study.

The trend with SST and female shark sightings in Gansbaai during this study was not statistically significant. It is possible that immature female white sharks prefer coastal aggregation sites in warmer conditions (El Niño phase of the SOI) to augment their own growth rate. In order to reach sexual maturity, a female white shark must attain a larger size than male sharks, approximately 4.5 m TL [77]. Investing less energy into thermoregulation by seeking out warmer more stable areas could provide more scope for growth, even though they can tolerate extremely low temperatures [10], [18], [21].

The results of this study showed that MEI had a significant effect on white sharks with the combined data set (males, females and unknown sex) in Gansbaai. It has become clear that climatic conditions exert powerful effects on fish stocks; with most variability occurring not annually but rather at decadal scales [78]. It is possible that the MEI influences the sexual composition of white sharks in Gansbaai and, presumably, other parts of their range. In this study, more females were seen in Gansbaai overall in warmer water/positive MEI years. Conversely, the opposite trend was observed for males. In cool water years (2010 to 2011) sightings of male sharks were significantly higher than in previous years. The same trend has been observed in the Neptune Islands where male sharks favour cool water conditions, and were more common than in warm water years [32], [33]. One explanation the authors propose for the greater abundance of males in cooler water years is the absence of larger females, which may reduce competition for prey resources. Competitive exclusion by larger conspecifics may occur at white shark aggregations where larger more experienced sharks occupy prime hunting areas excluding smaller less experienced sharks [79]. Sexual segregation has also been observed in white sharks at other locations [19], [32]. Another explanation could be that females may time their visits to areas when fewer males are around, or seek out inshore areas to avoid mating harassment [80]. MEI linked trends with white shark population composition may not have been demonstrated in other aggregation areas due to their shorter sampling periods [19]. For example, in South Australia, Bruce [81] and Malcolm et al. [82] reported a sex ratio bias towards female white sharks in the Dangerous Reef areas. This study was then contradicted by Robbins and Booth [32] who reported on a sex ratio bias towards males in 2003 to 2007 with a larger and more consistent data set in the same region. Similarly, in Gansbaai, earlier studies reported female biased population composition of white sharks with an overall paucity of males, particularly in summer months [52], [83], although these studies were limited to Dyer Island only. One similarity between these earlier studies is the timing in the early and late 1990s when El Niño conditions had impacts on various pelagic fish stocks worldwide, from anchovy and sardine to salmon and tuna [37], [78]. During 2003 and again in 2010, La Niña (cold water conditions) were in place, the latter being one of the most intense events in 50 years [37], (Figure 4).

Two previous studies have investigated the possible effects of ENSO on white sharks and their distribution. Martin [84] found no correlation between ENSO index and the number of white shark strandings from reports, photographs and fishing records in the northern latitude regions of the Pacific Ocean. He concluded that if anything, there was a slight positive, but not significant, correlation with La Niña (cooler SST) years. Similarly, Cliff et al. [49] investigated trends in catch per unit effort of the shark nets in KwaZulu-Natal and found a slight increase in inshore catches during La Niña years. Cooler upwelled waters were proposed as conditions that may possibly favour juvenile sharks in this subtropical part of the South African coast.

Even though white sharks are able to tolerate large changes in temperature while diving, [8], [10], [16], [18], [19], [21] the relationship between the white shark’s spatial and temporal distribution and water temperature is complex. Different size and age classes of white sharks are likely to have different tolerances to their physical environment, as well as different prey requirements [30], [85] and this will probably influence their movements. Similar to other marine predators, different sizes and sexes of white sharks prefer particular coastal regions during specific environmental conditions [32], [33], [78], [86]. Even though juvenile and sub adult white sharks do not face many threats from other marine predators, utilising the coastal regions at a time when larger sharks are less abundant could be favourable for their hunting as well as reducing predation risk. These periods of favourable environmental conditions (often termed cyclical loopholes or optimum environmental windows) are correlated with Southern Oscillation phenomena and have been discussed in detail by Bakun and Broad [78] who compared a wide variety of marine fishes and vertebrates with similar peaks and declines correlated with MEI. Furthermore, sub adult white sharks are partly piscivorous [85] and negative MEI conditions (cooler La Niña induced) are associated with increased productivity (largely linked to the increased upwelling of nutrient rich waters) which could perhaps have increased the availability of fish prey species resulting in more sub adult white sharks being attracted to the area.

The trend of increasing shark sightings over this study period has also been recorded in other areas where shark ecotourism is established [87]–[90]. This trend was not supported in a recent population estimate for the Gansbaai region as that study indicated that there was no increase in sharks over the last decade [90]. Bruce and Bradford [91] demonstrated extended residency periods of white sharks at an Australian study site where chumming and tourism activities expanded. Bait attracted sharks do have the potential to show what is described as ‘trap happy’ or ‘trap shy’ behaviour over time [89], [90]. Shark cage diving in South Africa, which had started in the 1980s, rapidly expanded in the 1990s. In 1991, white sharks were given national protected status. The coincidence of these events complicates the interpretation of the increasing trend found here. While it may be possible that chumming and diving activities have changed their behaviour and residence times [91] the opposite may also be true [89]. This issue requires further research in the future.

Previous work in South Africa has suggested that white shark inshore habitat use may be influenced by a seasonal migration of fish species inshore [33], [55]. The results of this study indicate that environmental influences may play a more important role in white shark abundance than hitherto thought, and should be addressed when drafting management plans for swimmer safety and white shark management. It may be possible to create a predictive model for risk of shark/human interaction once these variables are better understood. This could be of considerable benefit to reduce the risk of attack by sharks on humans in the Western Cape. This study has shown that knowledge of environmental variables contributes to a better understanding of the dynamic relationship between this apex predator and its habitat. Improved knowledge of the relative abundance and availability of their principal prey species could further enhance this understanding.
